# Poly[(μ-3,5-dinitro­benzoato)(μ-3,5-dinitro­benzoic acid)rubidium]

**DOI:** 10.1107/S160053681102513X

**Published:** 2011-07-06

**Authors:** Yanqing Miao, Tao Fan

**Affiliations:** a Xi’an Medical University, Department of Pharmacy, Hanguang Road No.137, Xi’an 710021, Shaanxi, People’s Republic of China

## Abstract

The asymmetric unit of the title compound, [Rb(C_7_H_3_N_2_O_6_)(C_7_H_4_N_2_O_6_)]_*n*_, comprises an Rb^+^ cation, a 3,5-dinitro­benzoate anion and a 3,5-dinitro­benzoic acid ligand. The Rb^+^ cation is nine-coordinated by O atoms from four 3,5-dinitro­benzoate anions and three neutral 3,5-dinitro­benzoic acid ligands. The metal atom is firstly linked by four bridging carboxyl groups, forming a binuclear motif, which is further linked by the nitro groups into a two-dimensional framework along the [110] direction. A short O—H⋯O hydrogen bond between two adjacent carboxy/carboxylate groups occurs.

## Related literature

For 3,5-dinitro­benzoate complexes, see: Askarinejad *et al.* (2007 ▶); Madej *et al.* (2007 ▶); Zhu *et al.* (2001 ▶). For Rb—O bond lengths, see: Cametti *et al.* (2005 ▶).
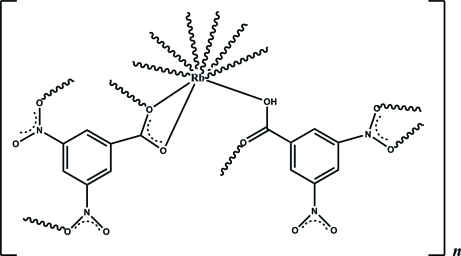


## Experimental

### 

#### Crystal data

[Rb(C_7_H_3_N_2_O_6_)(C_7_H_4_N_2_O_6_)]
*M* *_r_* = 508.71
Triclinic, 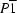

*a* = 9.4823 (8) Å
*b* = 9.8136 (8) Å
*c* = 11.4929 (11) Å
α = 68.425 (1)°
β = 83.821 (1)°
γ = 67.538 (1)°
*V* = 918.42 (14) Å^3^
*Z* = 2
Mo *K*α radiation
μ = 2.77 mm^−1^
*T* = 293 K
0.40 × 0.31 × 0.20 mm

#### Data collection

Bruker SMART CCD diffractometer
Absorption correction: multi-scan (*SADABS*; Sheldrick, 1996 ▶) *T*_min_ = 0.368, *T*_max_ = 0.635
4661 measured reflections
3219 independent reflections
2758 reflections with *I* > 2σ(*I*)
*R*_int_ = 0.015

#### Refinement

*R*[*F* ^2^ > 2σ(*F* ^2^)] = 0.030
*wR*(*F* ^2^) = 0.079
*S* = 1.08
3219 reflections
284 parameters
H atoms treated by a mixture of independent and constrained refinement
Δρ_max_ = 0.33 e Å^−3^
Δρ_min_ = −0.38 e Å^−3^

### 

Data collection: *SMART* (Bruker, 2002 ▶); cell refinement: *SAINT* (Bruker, 2002 ▶); data reduction: *SAINT*; program(s) used to solve structure: *SHELXS97* (Sheldrick, 2008 ▶); program(s) used to refine structure: *SHELXL97* (Sheldrick, 2008 ▶); molecular graphics: *SHELXTL* (Sheldrick, 2008 ▶); software used to prepare material for publication: *SHELXTL*.

## Supplementary Material

Crystal structure: contains datablock(s) I, global. DOI: 10.1107/S160053681102513X/aa2010sup1.cif

Structure factors: contains datablock(s) I. DOI: 10.1107/S160053681102513X/aa2010Isup2.hkl

Additional supplementary materials:  crystallographic information; 3D view; checkCIF report

## Figures and Tables

**Table 1:** Hydrogen-bond geometry (Å, °)

*D*—H⋯*A*	*D*—H	H⋯*A*	*D*⋯*A*	*D*—H⋯*A*
O7—H1⋯O1	0.96 (4)	1.52 (4)	2.470 (2)	168 (4)

**Fig. 1.**
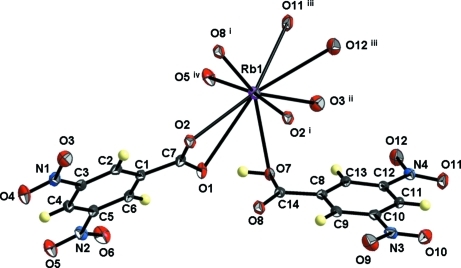
The structure of (I), showing the atomic numbering scheme. Non-H atoms are shown with 30% probability displacement ellipsoids. Symmetry codes: (i) 1 - x, 1 - y, 1 - z; (ii) 1 + x, - 1 + y, z; (iii) 2 - x, - y, 1 - z; (iv) 1 - x, 1 - y, 1 - z.

**Fig. 2.**
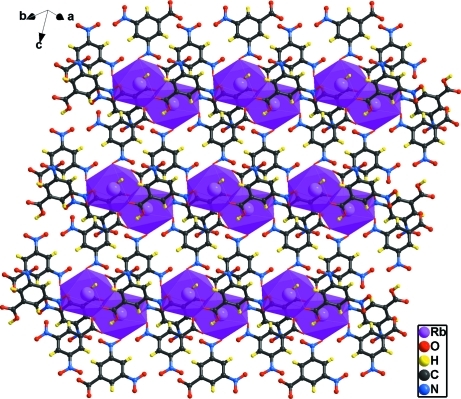
The two-dimensional framework of (I).

## supplementary crystallographic information

### Comment

The 3,5-dinitrobenzoic acid is an interesting ligand with one carboxylic and two nitro groups for coordination. In the structural investigation of 3,5-dinitrobenzoic acid complexes, it has been found that the 3,5-dinitrobenzoate moiety functions as a multidentate ligand (Askarinejad *et al.*, 2007; Madej *et al.*, 2007; Zhu *et al.*, 2001) with versatile binding and coordination modes. In this paper, we report the crystal structure of the title compound, a new Rb complex obtained by the reaction of 3,5-dinitrobenzoic acid and RbOH in water.

The asymmetric unit of the title compound (I) comprises a Rb^+^ cation, a 3,5-dinitrobenzoate anion and a 3,5-dinitrobenzoic acid ligand (Fig. 1). The central cation is coordinated to nine O atoms from four 3,5-dinitrobenzoate anions and three neutral 3,5-dinitrobenzoic acid ligands with the Rb—O distances ranging from 2.7973 (19) Å to 3.403 (2) Å, which are well within the range reported in the literature (Cametti *et al.*, 2005). The Rb centre is firstly linked by four bridging carboxylic groups to form a binuclear motif, which is further linked by the nitro groups to give the two-dimensional framework of the title compound (Fig. 2).

### Experimental

Analysis grade 3,5-dinitrobenzoic acid and RbOH (purity > 99.5%, Sinopharm Chemical Reagent Co., Ltd., Shanghai, China) were commercially available and used without further purification. To a solution of 20 mmol 3,5-dinitrobenzoic acid in 50 ml bidistilled water, a solution of 10 mmol RbOH in 40 ml bidistilled water was added dropwise at room temperature. After vigorous stirring for 3 h, the resulting solution was then evaporated to a volume of about 20 ml in vacuum and filtered hot. The filtrate was then set aside for crystallization at room temperature. Two weeks later, yellow block crystals of the title compound suitable for X-ray determination were isolated.

### Refinement

Carbon-bound H atoms were placed at calculated positions and were treated as riding on the parent C atoms with C – H = 0.93 Å, and with *U*_iso_(H) = 1.2 *U*_eq_(C). Oxygen-bound H atom was tentatively located in difference Fourier maps and was refined independently.

### Crystal data

**Table:**

[Rb(C_7_H_3_N_2_O_6_)(C_7_H_4_N_2_O_6_)]	*Z* = 2
*M**_r_* = 508.71	*F*(000) = 504
Triclinic, *P*1	*D*_x_ = 1.840 Mg m^−^^3^
Hall symbol: -P 1	Mo *K*α radiation, λ = 0.71073 Å
*a* = 9.4823 (8) Å	Cell parameters from 2183 reflections
*b* = 9.8136 (8) Å	θ = 2.3–25.6°
*c* = 11.4929 (11) Å	µ = 2.77 mm^−^^1^
α = 68.425 (1)°	*T* = 293 K
β = 83.821 (1)°	Block, yellow
γ = 67.538 (1)°	0.40 × 0.31 × 0.20 mm
*V* = 918.42 (14) Å^3^	

### Data collection

**Table:**

Bruker SMART CCD diffractometer	3219 independent reflections
Radiation source: fine-focus sealed tube	2758 reflections with *I* > 2σ(*I*)
graphite	*R*_int_ = 0.015
phi and ω scans	θ_max_ = 25.1°, θ_min_ = 1.9°
Absorption correction: multi-scan (*SADABS*; Sheldrick, 1996)	*h* = −11→11
*T*_min_ = 0.368, *T*_max_ = 0.635	*k* = −10→11
4661 measured reflections	*l* = −13→12

### Refinement

**Table:**

Refinement on *F*^2^	Primary atom site location: structure-invariant direct methods
Least-squares matrix: full	Secondary atom site location: difference Fourier map
*R*[*F*^2^ > 2σ(*F*^2^)] = 0.030	Hydrogen site location: inferred from neighbouring sites
*wR*(*F*^2^) = 0.079	H atoms treated by a mixture of independent and constrained refinement
*S* = 1.08	*w* = 1/[σ^2^(*F*_o_^2^) + (0.0471*P*)^2^ + 0.0239*P*] where *P* = (*F*_o_^2^ + 2*F*_c_^2^)/3
3219 reflections	(Δ/σ)_max_ = 0.001
284 parameters	Δρ_max_ = 0.33 e Å^−^^3^
0 restraints	Δρ_min_ = −0.38 e Å^−^^3^

### Special details

**Table:**

Geometry. All s.u.'s (except the s.u. in the dihedral angle between two l.s. planes) are estimated using the full covariance matrix. The cell s.u.'s are taken into account individually in the estimation of s.u.'s in distances, angles and torsion angles; correlations between s.u.'s in cell parameters are only used when they are defined by crystal symmetry. An approximate (isotropic) treatment of cell s.u.'s is used for estimating s.u.'s involving l.s. planes.
Refinement. Refinement of *F*^2^ against ALL reflections. The weighted *R*-factor *wR* and goodness of fit *S* are based on *F*^2^, conventional *R*-factors *R* are based on *F*, with *F* set to zero for negative *F*^2^. The threshold expression of *F*^2^ > σ(*F*^2^) is used only for calculating *R*-factors(gt) *etc*. and is not relevant to the choice of reflections for refinement. *R*-factors based on *F*^2^ are statistically about twice as large as those based on *F*, and *R*- factors based on ALL data will be even larger.

### Fractional atomic coordinates and isotropic or equivalent isotropic displacement parameters (Å^2^)

**Table:**

	*x*	*y*	*z*	*U*_iso_*/*U*_eq_	
Rb1	0.60363 (3)	0.23726 (3)	0.63137 (2)	0.04777 (12)	
O1	0.6222 (2)	0.5041 (3)	0.74637 (18)	0.0566 (5)	
O2	0.4151 (2)	0.5791 (2)	0.62901 (18)	0.0501 (5)	
O3	−0.0505 (2)	0.9948 (3)	0.6899 (2)	0.0726 (7)	
O4	−0.0484 (3)	1.1013 (3)	0.8223 (2)	0.0801 (7)	
O5	0.3968 (3)	0.9004 (3)	1.0902 (2)	0.0794 (7)	
O6	0.5892 (3)	0.6992 (3)	1.0817 (2)	0.0817 (7)	
O7	0.7938 (2)	0.4276 (2)	0.58487 (18)	0.0465 (4)	
H1	0.717 (5)	0.463 (5)	0.640 (4)	0.108 (14)*	
O8	0.6987 (2)	0.6736 (2)	0.44553 (19)	0.0532 (5)	
O9	0.9040 (3)	0.7679 (3)	0.0193 (2)	0.0801 (7)	
O10	1.1097 (3)	0.5933 (3)	−0.0103 (2)	0.0751 (7)	
O11	1.3434 (3)	0.1010 (3)	0.3122 (2)	0.0779 (7)	
O12	1.2396 (3)	0.0396 (3)	0.4887 (2)	0.0705 (6)	
C1	0.3944 (3)	0.6901 (3)	0.7846 (2)	0.0369 (5)	
C2	0.2455 (3)	0.7921 (3)	0.7486 (2)	0.0382 (6)	
H2	0.1956	0.7907	0.6839	0.046*	
C3	0.1717 (3)	0.8963 (3)	0.8101 (2)	0.0402 (6)	
C4	0.2396 (3)	0.9027 (3)	0.9064 (2)	0.0443 (6)	
H3	0.1888	0.9745	0.9461	0.053*	
C5	0.3874 (3)	0.7969 (3)	0.9414 (2)	0.0403 (6)	
C6	0.4654 (3)	0.6916 (3)	0.8829 (2)	0.0385 (6)	
H4	0.5650	0.6222	0.9091	0.046*	
C7	0.4807 (3)	0.5831 (3)	0.7125 (2)	0.0423 (6)	
C8	0.9000 (3)	0.4805 (3)	0.3865 (2)	0.0366 (5)	
C9	0.8963 (3)	0.5842 (3)	0.2643 (2)	0.0407 (6)	
H5	0.8216	0.6852	0.2370	0.049*	
C10	1.0068 (3)	0.5329 (3)	0.1847 (2)	0.0418 (6)	
C11	1.1200 (3)	0.3858 (3)	0.2214 (2)	0.0425 (6)	
H6	1.1936	0.3540	0.1666	0.051*	
C12	1.1197 (3)	0.2878 (3)	0.3423 (2)	0.0387 (6)	
C13	1.0115 (3)	0.3310 (3)	0.4255 (2)	0.0381 (6)	
H7	1.0136	0.2606	0.5066	0.046*	
C14	0.7854 (3)	0.5361 (3)	0.4767 (3)	0.0411 (6)	
N1	0.0124 (3)	1.0057 (3)	0.7712 (2)	0.0519 (6)	
N2	0.4643 (3)	0.7987 (3)	1.0451 (2)	0.0549 (6)	
N3	1.0066 (3)	0.6400 (3)	0.0554 (2)	0.0546 (6)	
N4	1.2440 (3)	0.1311 (3)	0.3850 (2)	0.0516 (6)	

### Atomic displacement parameters (Å^2^)

**Table:**

	*U*^11^	*U*^22^	*U*^33^	*U*^12^	*U*^13^	*U*^23^
Rb1	0.04757 (17)	0.04046 (16)	0.04939 (18)	−0.00983 (12)	−0.00575 (11)	−0.01423 (12)
O1	0.0394 (10)	0.0773 (15)	0.0520 (12)	−0.0082 (10)	0.0055 (9)	−0.0366 (11)
O2	0.0509 (11)	0.0616 (12)	0.0454 (11)	−0.0191 (10)	0.0037 (9)	−0.0297 (10)
O3	0.0460 (12)	0.0759 (16)	0.0887 (17)	−0.0075 (11)	−0.0183 (11)	−0.0315 (14)
O4	0.0614 (14)	0.0672 (15)	0.0889 (18)	0.0102 (12)	−0.0024 (12)	−0.0369 (14)
O5	0.0976 (18)	0.0906 (18)	0.0631 (15)	−0.0270 (15)	0.0006 (13)	−0.0492 (14)
O6	0.0751 (16)	0.0908 (19)	0.0759 (17)	−0.0125 (15)	−0.0291 (13)	−0.0365 (14)
O7	0.0445 (10)	0.0532 (12)	0.0453 (11)	−0.0170 (9)	0.0116 (9)	−0.0251 (10)
O8	0.0423 (10)	0.0490 (12)	0.0665 (13)	−0.0068 (10)	0.0019 (9)	−0.0293 (10)
O9	0.113 (2)	0.0466 (14)	0.0556 (14)	−0.0148 (14)	−0.0025 (13)	−0.0045 (11)
O10	0.1009 (18)	0.0685 (15)	0.0537 (14)	−0.0412 (14)	0.0285 (13)	−0.0160 (12)
O11	0.0647 (14)	0.0471 (13)	0.0999 (19)	−0.0078 (11)	0.0371 (13)	−0.0251 (12)
O12	0.0735 (15)	0.0445 (12)	0.0622 (14)	−0.0041 (11)	0.0064 (11)	−0.0044 (11)
C1	0.0396 (13)	0.0394 (14)	0.0306 (13)	−0.0163 (11)	0.0077 (10)	−0.0113 (11)
C2	0.0401 (14)	0.0439 (15)	0.0319 (13)	−0.0202 (12)	0.0038 (10)	−0.0107 (11)
C3	0.0370 (13)	0.0360 (14)	0.0389 (14)	−0.0106 (11)	0.0036 (11)	−0.0076 (11)
C4	0.0529 (16)	0.0398 (15)	0.0389 (15)	−0.0154 (13)	0.0085 (12)	−0.0164 (12)
C5	0.0459 (15)	0.0440 (15)	0.0310 (13)	−0.0175 (12)	0.0036 (11)	−0.0130 (11)
C6	0.0348 (13)	0.0448 (15)	0.0316 (13)	−0.0128 (12)	0.0035 (10)	−0.0114 (11)
C7	0.0425 (15)	0.0494 (16)	0.0353 (14)	−0.0182 (13)	0.0079 (11)	−0.0159 (12)
C8	0.0341 (12)	0.0391 (14)	0.0417 (14)	−0.0148 (11)	0.0015 (10)	−0.0188 (12)
C9	0.0399 (14)	0.0374 (14)	0.0460 (15)	−0.0111 (12)	−0.0031 (11)	−0.0182 (12)
C10	0.0515 (15)	0.0395 (14)	0.0374 (14)	−0.0224 (13)	0.0017 (12)	−0.0111 (11)
C11	0.0454 (14)	0.0424 (15)	0.0454 (15)	−0.0203 (13)	0.0146 (12)	−0.0211 (12)
C12	0.0372 (13)	0.0319 (13)	0.0478 (15)	−0.0125 (11)	0.0048 (11)	−0.0161 (11)
C13	0.0411 (13)	0.0380 (14)	0.0384 (14)	−0.0187 (12)	0.0040 (11)	−0.0137 (11)
C14	0.0314 (13)	0.0476 (16)	0.0524 (17)	−0.0141 (12)	0.0014 (11)	−0.0271 (14)
N1	0.0431 (13)	0.0441 (14)	0.0543 (15)	−0.0085 (11)	0.0039 (11)	−0.0105 (12)
N2	0.0658 (17)	0.0629 (17)	0.0408 (13)	−0.0243 (14)	−0.0014 (12)	−0.0223 (12)
N3	0.0771 (18)	0.0437 (15)	0.0466 (14)	−0.0293 (14)	0.0058 (13)	−0.0138 (12)
N4	0.0479 (14)	0.0381 (13)	0.0643 (17)	−0.0123 (11)	0.0093 (12)	−0.0192 (12)

### Geometric parameters (Å, °)

**Table:**

Rb1—O8^i^	2.7973 (19)	O12—N4	1.208 (3)
Rb1—O2^i^	2.853 (2)	O12—Rb1^iii^	3.274 (2)
Rb1—O7	2.9421 (19)	C1—C2	1.381 (3)
Rb1—O5^ii^	2.981 (2)	C1—C6	1.384 (4)
Rb1—O11^iii^	2.984 (2)	C1—C7	1.515 (3)
Rb1—O2	3.132 (2)	C2—C3	1.383 (4)
Rb1—O3^iv^	3.195 (2)	C2—H2	0.9300
Rb1—O12^iii^	3.274 (2)	C3—C4	1.371 (4)
Rb1—O1	3.403 (2)	C3—N1	1.479 (3)
O1—C7	1.284 (3)	C4—C5	1.381 (4)
O2—C7	1.218 (3)	C4—H3	0.9300
O2—Rb1^i^	2.853 (2)	C5—C6	1.373 (4)
O3—N1	1.217 (3)	C5—N2	1.472 (3)
O3—Rb1^v^	3.195 (2)	C6—H4	0.9300
O4—N1	1.215 (3)	C8—C13	1.379 (3)
O5—N2	1.223 (3)	C8—C9	1.394 (4)
O5—Rb1^ii^	2.981 (2)	C8—C14	1.508 (3)
O6—N2	1.206 (3)	C9—C10	1.385 (4)
O7—C14	1.292 (3)	C9—H5	0.9300
O7—H1	0.96 (4)	C10—C11	1.371 (4)
O8—C14	1.219 (3)	C10—N3	1.471 (3)
O8—Rb1^i^	2.7973 (19)	C11—C12	1.369 (4)
O9—N3	1.210 (3)	C11—H6	0.9300
O10—N3	1.220 (3)	C12—C13	1.379 (3)
O11—N4	1.215 (3)	C12—N4	1.476 (3)
O11—Rb1^iii^	2.984 (2)	C13—H7	0.9300
			
O8^i^—Rb1—O2^i^	74.83 (6)	C3—C2—H2	120.5
O8^i^—Rb1—O7	130.02 (6)	C4—C3—C2	122.9 (2)
O2^i^—Rb1—O7	70.65 (5)	C4—C3—N1	118.1 (2)
O8^i^—Rb1—O5^ii^	104.14 (7)	C2—C3—N1	119.0 (2)
O2^i^—Rb1—O5^ii^	169.95 (6)	C3—C4—C5	116.3 (2)
O7—Rb1—O5^ii^	103.80 (6)	C3—C4—H3	121.8
O8^i^—Rb1—O11^iii^	90.51 (7)	C5—C4—H3	121.8
O2^i^—Rb1—O11^iii^	114.53 (6)	C6—C5—C4	122.9 (2)
O7—Rb1—O11^iii^	136.55 (6)	C6—C5—N2	118.9 (2)
O5^ii^—Rb1—O11^iii^	75.35 (7)	C4—C5—N2	118.2 (2)
O8^i^—Rb1—O2	70.73 (5)	C5—C6—C1	119.3 (2)
O2^i^—Rb1—O2	78.03 (5)	C5—C6—H4	120.4
O7—Rb1—O2	67.61 (5)	C1—C6—H4	120.4
O5^ii^—Rb1—O2	92.15 (6)	O2—C7—O1	125.6 (2)
O11^iii^—Rb1—O2	154.45 (6)	O2—C7—C1	119.8 (2)
O8^i^—Rb1—O3^iv^	152.21 (6)	O1—C7—C1	114.6 (2)
O2^i^—Rb1—O3^iv^	105.72 (6)	C13—C8—C9	120.1 (2)
O7—Rb1—O3^iv^	73.40 (6)	C13—C8—C14	120.6 (2)
O5^ii^—Rb1—O3^iv^	79.97 (7)	C9—C8—C14	119.2 (2)
O11^iii^—Rb1—O3^iv^	63.58 (7)	C10—C9—C8	118.2 (2)
O2—Rb1—O3^iv^	137.02 (6)	C10—C9—H5	120.9
O8^i^—Rb1—O12^iii^	97.66 (6)	C8—C9—H5	120.9
O2^i^—Rb1—O12^iii^	78.90 (6)	C11—C10—C9	122.9 (2)
O7—Rb1—O12^iii^	109.61 (6)	C11—C10—N3	117.6 (2)
O5^ii^—Rb1—O12^iii^	111.08 (6)	C9—C10—N3	119.5 (2)
O11^iii^—Rb1—O12^iii^	39.61 (6)	C12—C11—C10	117.0 (2)
O2—Rb1—O12^iii^	156.22 (6)	C12—C11—H6	121.5
O3^iv^—Rb1—O12^iii^	56.26 (6)	C10—C11—H6	121.5
O8^i^—Rb1—O1	108.63 (5)	C11—C12—C13	122.9 (2)
O2^i^—Rb1—O1	98.35 (5)	C11—C12—N4	117.8 (2)
O7—Rb1—O1	45.08 (5)	C13—C12—N4	119.3 (2)
O5^ii^—Rb1—O1	72.34 (6)	C12—C13—C8	118.9 (2)
O11^iii^—Rb1—O1	145.53 (6)	C12—C13—H7	120.6
O2—Rb1—O1	39.54 (5)	C8—C13—H7	120.6
O3^iv^—Rb1—O1	98.85 (6)	O8—C14—O7	126.1 (2)
O12^iii^—Rb1—O1	151.95 (5)	O8—C14—C8	120.0 (3)
C7—O1—Rb1	87.28 (15)	O7—C14—C8	113.8 (2)
C7—O2—Rb1^i^	127.37 (18)	O4—N1—O3	123.7 (3)
C7—O2—Rb1	101.53 (17)	O4—N1—C3	118.1 (3)
Rb1^i^—O2—Rb1	101.97 (5)	O3—N1—C3	118.2 (2)
N1—O3—Rb1^v^	112.76 (17)	O6—N2—O5	123.7 (3)
N2—O5—Rb1^ii^	111.6 (2)	O6—N2—C5	118.4 (2)
C14—O7—Rb1	119.78 (15)	O5—N2—C5	117.9 (3)
C14—O7—H1	113 (3)	O6—N2—Rb1^ii^	86.28 (17)
Rb1—O7—H1	74 (3)	O5—N2—Rb1^ii^	50.10 (15)
C14—O8—Rb1^i^	120.61 (16)	C5—N2—Rb1^ii^	137.66 (17)
N4—O11—Rb1^iii^	100.05 (17)	O9—N3—O10	123.6 (3)
N4—O12—Rb1^iii^	86.09 (16)	O9—N3—C10	118.7 (2)
C2—C1—C6	119.5 (2)	O10—N3—C10	117.7 (3)
C2—C1—C7	119.8 (2)	O12—N4—O11	123.8 (2)
C6—C1—C7	120.6 (2)	O12—N4—C12	118.5 (2)
C1—C2—C3	119.0 (2)	O11—N4—C12	117.7 (2)
C1—C2—H2	120.5		
			
O8^i^—Rb1—O1—C7	−4.89 (16)	Rb1—O1—C7—C1	155.4 (2)
O2^i^—Rb1—O1—C7	71.84 (15)	Rb1—O1—C7—Rb1^i^	−67.82 (12)
O7—Rb1—O1—C7	123.58 (17)	C2—C1—C7—O2	−5.5 (4)
O5^ii^—Rb1—O1—C7	−104.28 (16)	C6—C1—C7—O2	177.5 (2)
O11^iii^—Rb1—O1—C7	−125.39 (16)	C2—C1—C7—O1	173.8 (2)
O2—Rb1—O1—C7	12.30 (14)	C6—C1—C7—O1	−3.3 (4)
O3^iv^—Rb1—O1—C7	179.29 (15)	C2—C1—C7—Rb1^i^	36.6 (3)
O12^iii^—Rb1—O1—C7	153.88 (15)	C6—C1—C7—Rb1^i^	−140.5 (2)
N4^iii^—Rb1—O1—C7	−163.41 (14)	C13—C8—C9—C10	−0.1 (4)
C14^i^—Rb1—O1—C7	2.46 (15)	C14—C8—C9—C10	177.2 (2)
N2^ii^—Rb1—O1—C7	−92.20 (16)	C8—C9—C10—C11	−0.7 (4)
O8^i^—Rb1—O2—C7	149.51 (17)	C8—C9—C10—N3	−179.6 (2)
O2^i^—Rb1—O2—C7	−132.57 (18)	C9—C10—C11—C12	0.5 (4)
O7—Rb1—O2—C7	−58.77 (16)	N3—C10—C11—C12	179.4 (2)
O5^ii^—Rb1—O2—C7	45.28 (17)	C10—C11—C12—C13	0.5 (4)
O11^iii^—Rb1—O2—C7	104.7 (2)	C10—C11—C12—N4	−177.8 (2)
O3^iv^—Rb1—O2—C7	−32.3 (2)	C11—C12—C13—C8	−1.3 (4)
O12^iii^—Rb1—O2—C7	−146.79 (17)	N4—C12—C13—C8	177.0 (2)
O1—Rb1—O2—C7	−13.23 (15)	C9—C8—C13—C12	1.0 (4)
C14^i^—Rb1—O2—C7	154.64 (18)	C14—C8—C13—C12	−176.2 (2)
N2^ii^—Rb1—O2—C7	45.08 (16)	Rb1^i^—O8—C14—O7	−81.4 (3)
O8^i^—Rb1—O2—Rb1^i^	−77.92 (6)	Rb1^i^—O8—C14—C8	100.6 (2)
O2^i^—Rb1—O2—Rb1^i^	0.0	Rb1—O7—C14—O8	89.7 (3)
O7—Rb1—O2—Rb1^i^	73.80 (6)	Rb1—O7—C14—C8	−92.2 (2)
O5^ii^—Rb1—O2—Rb1^i^	177.84 (7)	Rb1—O7—C14—Rb1^i^	44.33 (18)
O11^iii^—Rb1—O2—Rb1^i^	−122.72 (14)	C13—C8—C14—O8	171.6 (2)
O3^iv^—Rb1—O2—Rb1^i^	100.29 (9)	C9—C8—C14—O8	−5.6 (4)
O12^iii^—Rb1—O2—Rb1^i^	−14.22 (17)	C13—C8—C14—O7	−6.6 (3)
O1—Rb1—O2—Rb1^i^	119.33 (9)	C9—C8—C14—O7	176.2 (2)
C14^i^—Rb1—O2—Rb1^i^	−72.80 (7)	C13—C8—C14—Rb1^i^	−139.52 (19)
N2^ii^—Rb1—O2—Rb1^i^	177.65 (7)	C9—C8—C14—Rb1^i^	43.2 (3)
O8^i^—Rb1—O7—C14	−39.1 (2)	Rb1^v^—O3—N1—O4	−1.9 (4)
O2^i^—Rb1—O7—C14	9.82 (17)	Rb1^v^—O3—N1—C3	178.04 (16)
O5^ii^—Rb1—O7—C14	−161.43 (18)	C4—C3—N1—O4	3.4 (4)
O11^iii^—Rb1—O7—C14	115.44 (19)	C2—C3—N1—O4	−176.7 (3)
O2—Rb1—O7—C14	−74.83 (18)	C4—C3—N1—O3	−176.5 (3)
O3^iv^—Rb1—O7—C14	123.67 (18)	C2—C3—N1—O3	3.4 (4)
O12^iii^—Rb1—O7—C14	79.84 (18)	Rb1^ii^—O5—N2—O6	−48.8 (4)
O1—Rb1—O7—C14	−114.75 (19)	Rb1^ii^—O5—N2—C5	130.3 (2)
N4^iii^—Rb1—O7—C14	99.53 (18)	C6—C5—N2—O6	−5.8 (4)
C14^i^—Rb1—O7—C14	−45.6 (2)	C4—C5—N2—O6	174.8 (3)
N2^ii^—Rb1—O7—C14	−144.50 (18)	C6—C5—N2—O5	175.1 (3)
C6—C1—C2—C3	1.4 (4)	C4—C5—N2—O5	−4.3 (4)
C7—C1—C2—C3	−175.7 (2)	C6—C5—N2—Rb1^ii^	−124.7 (2)
C1—C2—C3—C4	−0.3 (4)	C4—C5—N2—Rb1^ii^	55.9 (4)
C1—C2—C3—N1	179.8 (2)	C11—C10—N3—O9	176.9 (3)
C2—C3—C4—C5	−0.9 (4)	C9—C10—N3—O9	−4.2 (4)
N1—C3—C4—C5	178.9 (2)	C11—C10—N3—O10	−2.2 (4)
C3—C4—C5—C6	1.1 (4)	C9—C10—N3—O10	176.8 (3)
C3—C4—C5—N2	−179.5 (2)	Rb1^iii^—O12—N4—O11	−32.7 (3)
C4—C5—C6—C1	−0.1 (4)	Rb1^iii^—O12—N4—C12	145.8 (2)
N2—C5—C6—C1	−179.4 (2)	Rb1^iii^—O11—N4—O12	36.9 (3)
C2—C1—C6—C5	−1.2 (4)	Rb1^iii^—O11—N4—C12	−141.63 (19)
C7—C1—C6—C5	175.9 (2)	C11—C12—N4—O12	−175.2 (3)
Rb1^i^—O2—C7—O1	−86.6 (3)	C13—C12—N4—O12	6.5 (4)
Rb1—O2—C7—O1	28.4 (3)	C11—C12—N4—O11	3.4 (4)
Rb1^i^—O2—C7—C1	92.5 (3)	C13—C12—N4—O11	−174.9 (3)
Rb1—O2—C7—C1	−152.5 (2)	C11—C12—N4—Rb1^iii^	−72.2 (4)
Rb1—O2—C7—Rb1^i^	114.97 (17)	C13—C12—N4—Rb1^iii^	109.5 (3)
Rb1—O1—C7—O2	−25.4 (3)		

Symmetry codes: (i) −*x*+1, −*y*+1, −*z*+1; (ii) −*x*+1, −*y*+1, −*z*+2; (iii) −*x*+2, −*y*, −*z*+1; (iv) *x*+1, *y*−1, *z*; (v) *x*−1, *y*+1, *z*.

### Hydrogen-bond geometry (Å, °)

**Table:**

*D*—H···*A*	*D*—H	H···*A*	*D*···*A*	*D*—H···*A*
O7—H1···O1	0.96 (4)	1.52 (4)	2.470 (2)	168 (4)
